# Medication Errors in Pediatrics: Proposals to Improve the Quality and Safety of Care Through Clinical Risk Management

**DOI:** 10.3389/fmed.2021.814100

**Published:** 2022-01-14

**Authors:** Stefano D'Errico, Martina Zanon, Davide Radaelli, Martina Padovano, Alessandro Santurro, Matteo Scopetti, Paola Frati, Vittorio Fineschi

**Affiliations:** ^1^Department of Medicine, Surgery, and Health, University of Trieste, Trieste, Italy; ^2^Department of Anatomical, Histological, Forensic and Orthopaedic Sciences, Sapienza University of Rome, Rome, Italy; ^3^Department of Medicine, Surgery and Dentistry, University of Salerno, Salerno, Italy

**Keywords:** computerized prescription order entry, healthcare quality, patient safety, prescribing errors, malpractice litigation, medication errors

## Abstract

Medication errors represent one of the most common causes of adverse events in pediatrics and are widely reported in the literature. Despite the awareness that children are at increased risk for medication errors, little is known about the real incidence of the phenomenon. Most studies have focused on prescription, although medication errors also include transcription, dispensing, dosage, administration, and certification errors. Known risk factors for therapeutic errors include parenteral infusions, oral fluid administration, and tablet splitting, as well as the off-label use of drugs with dosages taken from adult literature. Emergency Departments and Intensive Care Units constitute the care areas mainly affected by the phenomenon in the hospital setting. The present paper aims to identify the risk profiles in pediatric therapy to outline adequate preventive strategies. Precisely, through the analysis of the available evidence, solutions such as standardization of recommended doses for children, electronic prescribing, targeted training of healthcare professionals, and implementation of reporting systems will be indicated for the prevention of medication errors.

## Introduction

Despite the progress achieved in recent years by pharmacological research in the pediatric population, the problem of the availability of drugs suitable for children has not yet been solved, and the percentage of drugs studied for pediatric age remains below 50% (1). The subject is even more important in the neonatology field, as some problems related to clinical trials in that age group are amplified both for ethical and economic reasons. Consequently, the use of adjusted adult dosage or the off-label drug administration in the pediatric population are frequent. Such use determines the exposure to increased risks of medication errors and adverse reactions.

Over the years, the scientific community has emphasized the need to raise awareness among the population on the responsible use of drugs in pediatrics and on the importance of promoting clinical studies in the field. The pediatric population is poorly investigated about the rational use of the drugs intended for it. Not all drugs used in pediatric settings have the same response in infants, children, and adolescents, due to differences in metabolism and absorption as well as different growth processes. Therefore, at the time of administration, a lot of attention must be paid to the choice of the medicines and the respective dosages, to be scrupulously evaluated based on the characteristics of the patient.

Medication errors are defined as any avoidable event in prescribing, transcribing, dispensing, administering, or monitoring, regardless of the occurrence of injury or potential injury; such events can result from human errors or system defects (2). The most frequently identified errors in pediatrics relate to the use of adult formulated medications and the need to change original dosages (3).

In such a composite scenario, the clinical risk management is fundamental as it allows to define risk profiles and plan governance strategies (4, 5). The systematic assessment of structural aspects and care pathways plays a significant role in identifying and reducing the risk of error. According to this perspective, the control of compliance with protocols, the correctness of communication processes, the adequacy of clinical records, and the training of health personnel are crucial (6). The American Academy of Pediatrics recommended in 2001 the adoption of reporting and learning systems to identify medication errors as a fundamental step to improve the quality and safety in pediatric care (7, 8).

The objective of the present paper is to outline the fundamental principles of risk management and strategic planning in the context of therapeutic errors in pediatrics. For this purpose, risk profiling will be carried out with a description of the main sources of therapeutic error in pediatric care. Consequently, reactive measures to minimize the risk of medication errors, as well as proactive approaches to strategic planning of care pathways, will be suggested.

## Materials and Methods

The present review was conducted by searching the PubMed database. The question was formulated using the PICO framework; in particular, to expand research and not to exclude potentially relevant articles, a calibration was carried out on pediatric patients and medication errors (“P,” patient/problem), drug therapy (“I,” intervention/treatment) and adverse events (“O,” outcome).

The “full text” and “English” filters have been applied. No time limits have been set. The last search was done on August 17, 2021. The PubMed “similar articles” section and the references of the selected articles were used to broaden the search. The screening included a first phase of analysis of the titles and abstracts as well as a second phase of review of the full texts.

Studies concerning epidemiology, setting (hospital and outpatient), type of adverse events, risk factors, and personalization of therapeutic pathways have been included. No exclusion criteria have been set.

## Epidemiology

### Inpatient Setting

**Figure 1:**
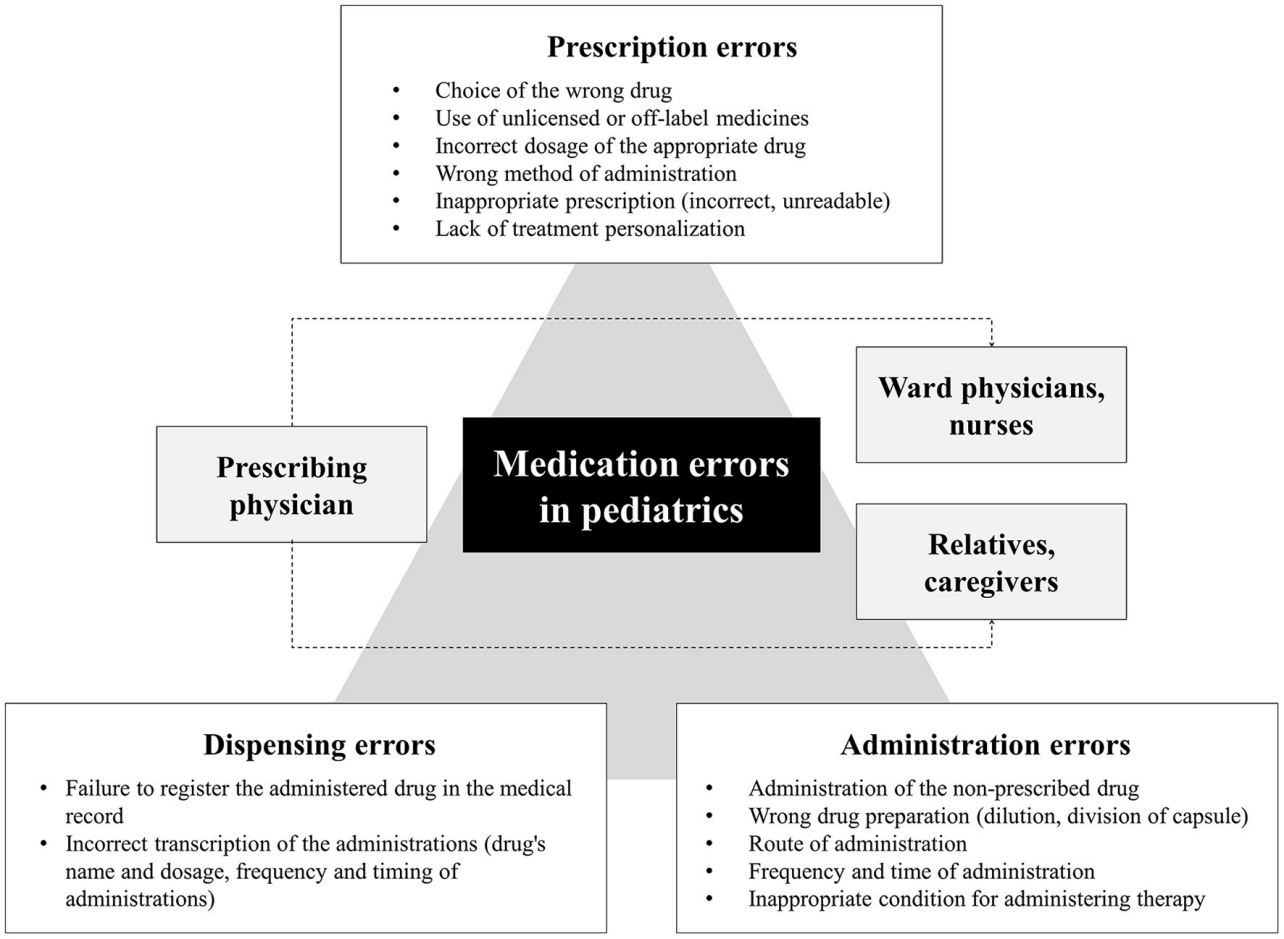
Classification of therapeutic errors based on the different phases of healthcare.

In the pediatric field, the administration of drugs involves a great responsibility, as it directly affects the safety and health of the assisted children. Despite medication errors in children are frequently reported, their epidemiology is difficult to completely understand. It has been estimated that 7.5 million preventable medication errors could occur with pediatric patients in the United States each year (9). Other studies estimated that 14–31% of pediatric medication errors could result in harm or death (10, 11). The US Pharmacopeia (USP) Medication Errors Reporting Program reported higher rates of medication errors in pediatric patients (31%) than in adults (13%) (12). According to some Authors, an error in the overall spectrum of the delivery process involving prescribing, dispensing, and administering was observed in 5–27% of medication orders for children (13) (Figure 1). Takata et al. observed a 22% of preventable medication error in the inpatient pediatric setting, which could have been identified earlier (17.8%) or mitigated more effectively (16.8%) (14).

Medication errors represent one of the main concerns in the inpatient setting, showing a prevalence for emergency, intensive care, and anesthesiology departments, where severe diseases and life-threatening conditions are frequent (15). Nichter et al. observed an incidence of medication errors in pediatric intensive care units ranging between 22 and 59 errors per 1,000 doses, seven times more frequently than other pediatric inpatient units (16). Furthermore, the reported risk of medication errors in neonatal wards is eight times higher than that of adults' ones (17, 18). An incidence ranging between 13 and 91 medication errors per 100 admissions in the Neonatal Intensive Care Unit (NICU) has been estimated with rates of occurrence considerably higher in preterm neonates (75.4 vs. 24.6%) (19). According to Burton et al., 60% of interviewed anesthesiologists reported pediatric medication errors at least once a year, and 15% of them experienced an error at least once a month (20). Similarly, emergency care represents a high-risk setting for therapeutic errors with a reported percentage between 10 and 31%; these events were more often dependent on errors in weight or unit of measurement, duplication of the dose, calculation errors, and choice of the wrong drug (21, 22).

The causes of medication errors are generally multifactorial. Dosage and administration errors remain the most reported inadequacies in inpatient settings, particularly in the case of unlicensed (7-10%) and off-label (18–64%) drug administration (23). Precisely, higher percentages of medication errors have been observed in off-label administration, antibiotic prescribing, and intravenous use (24–27). Furthermore, the use of unlicensed or off-label medicines observed in NICUs and Pediatric Intensive Care Units (PICUs), as well as sedation state, are reported as potential contributory factor to errors. Childhood obesity constitutes another opportunity for dosing errors (underdosing). Birth weight and prematurity require dosage and dose interval adjustments, making the prescriptive and administration phases susceptible to life-threatening errors. In addition, lacking standardized dosing regimens for children, most medication dosing requires calculations that increase the risk and impact of errors (28, 29). Preparation of the dilutions and the need to open the capsules to split them are indicated as further risk factors for administration errors. The risk of medication error can even be increased because of the availability of different preparations of the same drug combined with diverse formulations for pediatric and adult administration. Verbal prescriptions, frequent interruptions, and lack of clinical pharmacists in the care team are finally important risk factors for therapeutic errors (30).

According to Stratton et al. half of the interviewed pediatric nurses cited distraction as a crucial contributing factor (31); repetitive interruptions during drug preparation and fatigue are referred as to the most common causative factor (20). Insufficient knowledge of drugs and inaccurate reading of prescriptions were indicated as other significant causes of medication errors in association with unreadable handwriting of the prescriber. Heavy workload, insufficient communication with personnel, and availability of drugs with packaging or similar names (Look-Alike Sound-Alike, LASA) can also contribute to errors. Finally, nurses complained about lacking appropriate guidelines for drug administration to pediatric patients, as observed in other studies (32, 33).

### Outpatient Setting

Medication errors in the outpatient setting determine several emergency departments accesses annually (34). The real incidence of the phenomenon in the outpatient setting is however difficult to estimate and the data are generally underestimated. The main reason for the underestimation of outpatient treatment errors lies in the failure to communicate the problem that has occurred to the care provider, especially in cases where no harmful effects arise (35).

The most frequently reported issues include an incorrect dosage or frequency of administration, drug inadequacy for the disease, incorrect route of administration, misinterpretation of drug interactions, poor surveillance of side effects, and lack of communication (36). In a study of 147 outpatient errors (including 47 medication errors), 55% related to order, 30% to non-ordering, and 11% to administration (37). In a further study carried out on 1933 prescriptions, it was found that 15% of patients had a potential dosage error, with a higher frequency (33%) in children weighing <35 kg (38); a similar increase in incidence was found for subjects under the age of 4 and in cases of multiple prescriptions. The drug classes most involved in overdose cases include analgesics (15%), while in underdose cases antiepileptics (20%) represent the most implicated drugs.

## Preventive Strategies

In 2001, the Institute for Safe Medication Practices (ISMP) published guidelines for preventing medication errors in pediatrics recommending Computerized Provider Order Entry (CPOE), barcoding technology, unit-dose dispensing systems, and educational systems for healthcare professionals (39). Several authors experienced a significant reduction in prescribing and dose error rates with health information technology (40–42). Rinke et al. reported a considerable decrease in dosing errors during the implementation of CPOE in support of clinical decision making (43). Similarly, a reduction in prescription errors was observed with the use of pre-printed order sheets instead of the manual entry of the order. In any case, CPOE cannot be considered risk-free of errors, in fact, not all computerized systems calculate dosages or ensure that these do not exceed the maximum recommended according to weight and age (44). A review of information technology interventions revealed that only one-half of the randomized controlled trials studied demonstrated a significant reduction of medication errors with e-prescribing; moreover, low sensitivity and specificity of the alert systems were found (45).

Reduction of medication administration errors was observed with the implementation of Bar Code Medication Administration (BCMA) due to the improvement of accuracy indicators such as patient identity verification and charting after administration (46).

The competence of the healthcare personnel, the environment, the policies related to verbal orders, as well as clear and accurate drug labeling, are proposed as relatively inexpensive prevention tools (47).

Promoting a safety culture and awareness of medication errors between healthcare providers is mandatory in healthcare organizations. Prot et al. observed a reduction of medication errors after introducing regular briefing of pediatric nurses on safe medication administration and proper training of newly engaged nurses (23).

The Institute for Safe Medication Practices highlights the importance of voluntary error reporting to identify medication safety issues, system-related causes, and beneficial system changes that have been made as a result (48).

Parental training and quality improvement programs represent an alternative strategy for the prevention of dosing errors in outpatient settings. Relatives or caregivers of pediatric patients necessarily need to improve their skills and knowledge about the risk of dose dumping, and the erratic release of the active ingredient, thus modulating the effectiveness of the treatment (49).

**Figure 2:**
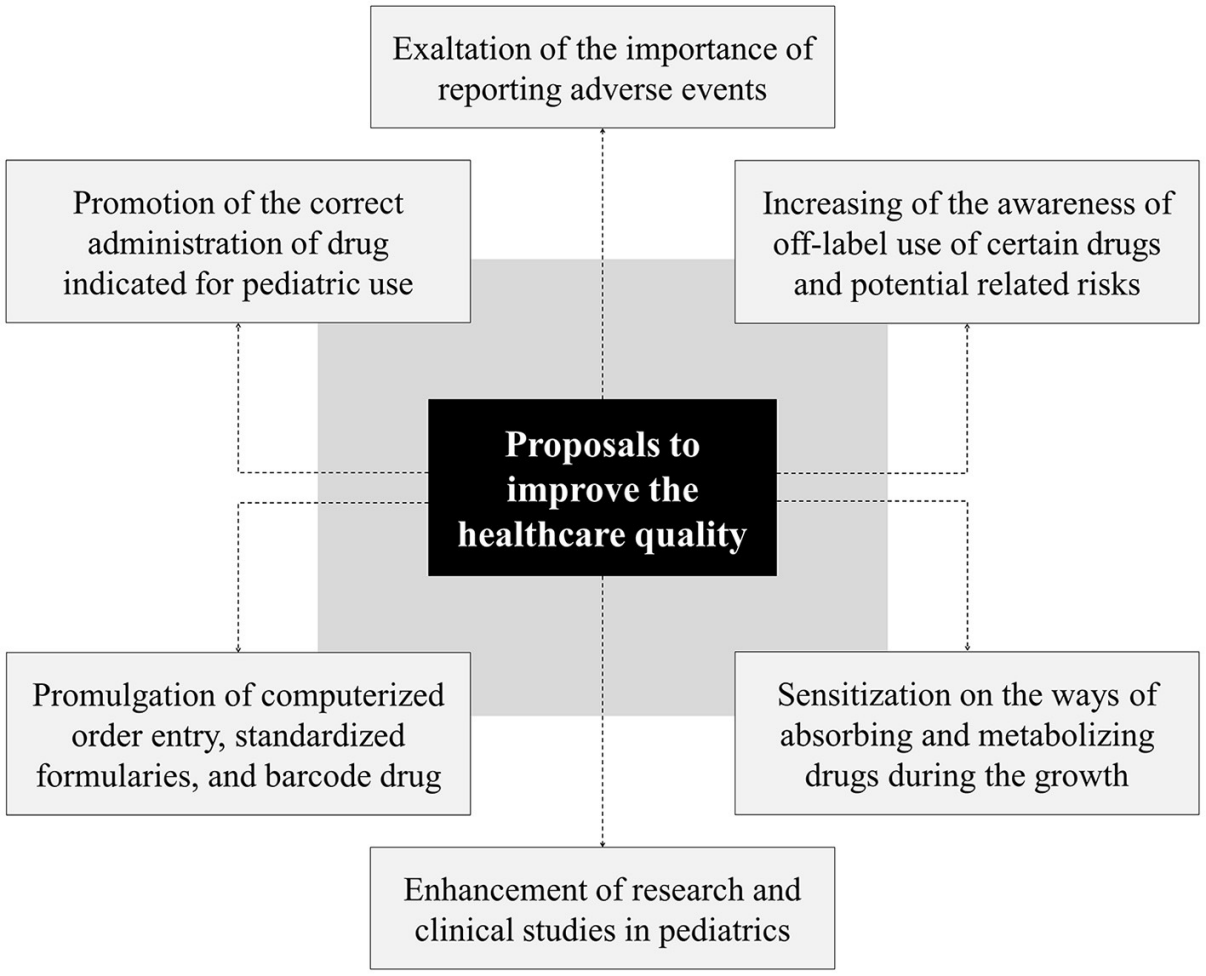
Preventive strategies to improve the quality and safety of care.

The literature data confirm the possibility of implementing multiple preventive strategies in the field of computerization, administration, reporting, and research. Summarizing the above, potential interventions aimed at reducing errors and improving pharmacologic pediatric treatments include (Figure 2):

◾ promotion of correct use of the drug in the pediatric field as well as sensitization of population and healthcare professionals on possible risks deriving from the administration of drugs authorized for adult use;
◾ increasing of the awareness of current off-label use of certain drugs and potential related problems;
◾ sensitization on the developmental variability of absorbing and metabolizing drugs;
◾ emphasizing the importance of reporting adverse events;
◾ information on the value of research and clinical studies to improve the quality and safety of pediatric drugs;
◾ implementation of computerized order entry, standardized formularies, barcode medication administration.

## Discussion

Patient safety represents one of the major challenges for modern healthcare systems due to the significant impact of adverse events in medicine. On the other hand, risk is an intrinsic component of health care since error is a possible event as it is related to the human component. Medication errors in pediatrics constitute a significant source of concern for health systems due to the implications in terms of quality of care, patient safety, and professional liability. The increase in the complexity of health interventions and the use of gradually more invasive procedures determine an exponential growth in the correlated risk, understood as the probability for the patient to experience an adverse event. In cases in which the adverse event is due to an error, it must be considered predictable and preventable through risk management strategies.

In the field of medication errors, risk management planning should be proactive and, therefore, based on a systematic analysis of care paths and therapeutic modalities. Indeed, there is considerable evidence that testifies how medication errors can occur at any stage of the treatment process, from prescription to storage, dispensing, preparation, and administration. In general, the error can manifest itself through over or underdosing, choosing an inappropriate route of administration, or administering the correct drug to the wrong patient. The consequences of medication error can be significant, sometimes fatal, and include an increase in the incidence and severity of adverse reactions and loss of drug efficacy.

The application of proactive risk management methods makes it possible to improve patient safety, to implement the ability to report critical issues, carry out risk mapping, as well as to plan optimization programs, and take corrective actions on areas at risk.

The analysis of the error must not have any inquisitive character but must consider the fallibility of health professionals and favor a global clinical approach for error prevention through understanding the causes. The identification of sources of risk should also include the study of all errors that have the potential to cause an adverse event that does not occur by chance or because it was intercepted; naturally, the decision on the risk class to be attributed to the phenomenon will depend on the incidence that can be inferred from the reporting systems.

Obviously, training is confirmed as an indispensable tool for inducing change and creating a culture of safety in all the involved operators. The strengthening of skills through activities centered on training needs promotes the awareness of health professionals on appropriateness, quality, and safety of care.

Healthcare organizations must investigate all the aspects of pediatric pharmacologic treatment and adopt effective interventions, and policies to reduce medication errors. Such an objective can be reached through research, implementation of surveillance systems, and personalization of pharmacological therapies (50–54). Furthermore, the consolidation of information technology and social sensitization are recommended.

Alongside the methodological limits in the application of the proactive approach, it should be noted that the subjective component also determines risk management problems, influencing the results based on the level of competence. Secondly, critical issues are too often analyzed individually and statically, while in health care adverse events result from multiple insufficiencies and often correlated conditions. Furthermore, the description of errors in some complex therapeutic pathways requires considerable synthesis skills on the part of data analysis professionals.

Conclusively, medication error causes repercussions on the quality of care and represents a serious threat to the safety of pediatric patients. Reports of literature are comfortable but additional research is needed to identify optimal strategies. It is therefore essential to develop a prevention-oriented risk culture based on the belief that errors if detected and analyzed in a correct manner, represent a precious opportunity for improvement (5, 55–57). Promoting safety and quality in healthcare providers and caregivers remains mandatory (58).

## Author Contributions

SD, VF, and PF contributed to conceptualization. AS and MS contributed to methodology and validation. MZ, DR, and MP performed investigation and formal analysis. SD, MP, and MS contributed to the original draft preparation. SD, PF, and VF contributed to supervision and final approval of the manuscript. All authors listed have made a substantial, direct, and intellectual contribution to the work and approved it for publication.

## Conflict of Interest

The authors declare that the research was conducted in the absence of any commercial or financial relationships that could be construed as a potential conflict of interest.

## Publisher's Note

All claims expressed in this article are solely those of the authors and do not necessarily represent those of their affiliated organizations, or those of the publisher, the editors and the reviewers. Any product that may be evaluated in this article, or claim that may be made by its manufacturer, is not guaranteed or endorsed by the publisher.
